# Dementia death rates prediction

**DOI:** 10.1186/s12888-023-05172-2

**Published:** 2023-09-22

**Authors:** Oleg Gaidai, Vladimir Yakimov, Rajiv Balakrishna

**Affiliations:** 1 Shanghai Ocean University, Shanghai, China; 2 Central Marine Research and Design Institute, Saint Petersburg, Russia; 3 University of Stavanger, Stavanger, Norway

**Keywords:** Dementia, AI, Mathematical biology, Risk forecast, Public health

## Abstract

**Background:**

Prevalence of dementia illness, causing certain morbidity and mortality globally, places burden on global public health. This study primary goal was to assess future risks of dying from severe dementia, given specific return period, within selected group of regions or nations.

**Methods:**

Traditional statistical approaches do not have benefits of effectively handling large regional dimensionality, along with nonlinear cross-correlations between various regional observations. In order to produce reliable long-term projections of excessive dementia death rate risks, this study advocates novel bio-system reliability technique, that being particularly suited for multi-regional environmental, biological, and health systems.

**Data:**

Raw clinical data has been used as an input to the suggested population-based, bio-statistical technique using data from medical surveys and several centers.

**Results:**

Novel spatiotemporal health system reliability methodology has been developed and applied to dementia death rates raw clinical data. Suggested methodology shown to be capable of dealing efficiently with spatiotemporal clinical observations of multi-regional nature. Accurate disease risks multi-regional spatiotemporal prediction being done, relevant confidence intervals have been presented as well.

**Conclusions:**

Based on available clinical survey dataset, the proposed approach may be applied in a variety of clinical public health applications. Confidence bands, given for predicted dementia-associated death rate levels with return periods of interest, have been reasonably narrow, indicating practical values of advocated prognostics.

## Key points

▪ Novel spatiotemporal health system reliability methodology has been developed and applied to dementia death rates raw clinical data.

▪ Accurate disease risks multi-regional spatiotemporal prediction being done.

▪ Confidence bands given for predicted dementia-associated death rate levels with return periods of interest.

## Introduction

**Fig. 1:**
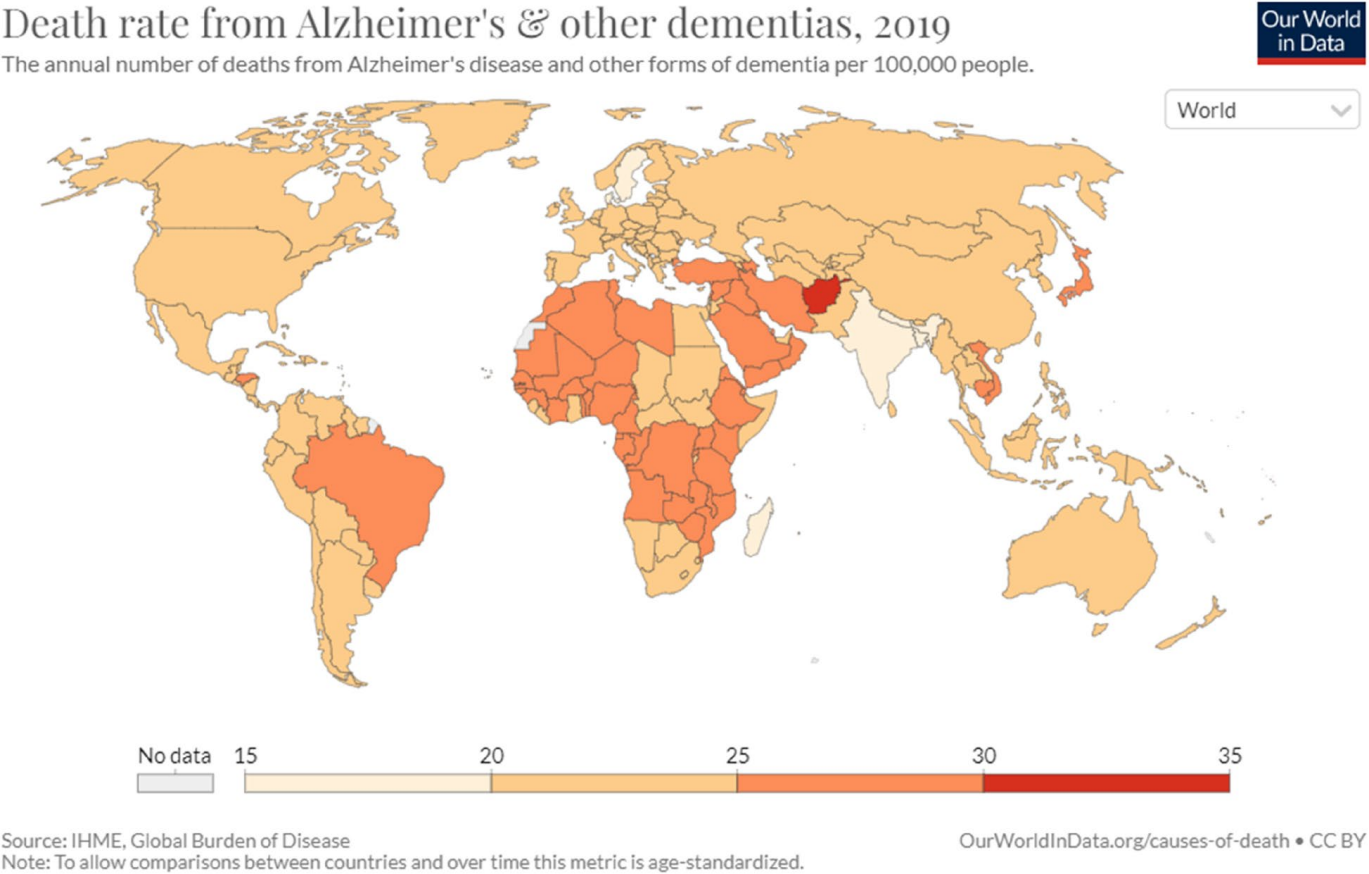
World’s countries map with dementia cases. All world’s countries have been accounted for in this study, (https://ourworldindata.org/causes-of-death)

WHO (World Health Organization) estimates that the number of individuals with dementia worldwide being approximately 55 million, and this number expected to reach about 80 million by 2030 and 140 million by 2050 [1]. Dementia trends more pronounced in South Korea, country experiencing rapid population aging; number of people aged 65, and older reached 8.5 million in 2021, and is expected to exceed 13 million by 2030, and 19 million (accounting for about 40% of older adult population) by 2050. There are several types of dementia, with Alzheimer's disease being the most prevalent. Alzheimer’s disease along with vascular dementia being the most common dementia forms; to mention other types, one can refer to Lewy body dementia, frontotemporal dementia and Parkinson’s disease with dementia. This condition causes cognitive function and capacity to decline more quickly, than would be expected with normal aging; it might manifest itself either chronically or gradually. Dementia diseases impair range of mental processes, including learning, memory, understanding, judgment, and language. Figure 1 displays global map, showing prevalence of dementia-related death rates. In this study these rates have been age-averaged, but accounting for spatial variations, given list of countries of interest, as well as relevant temporal variations. Statistical aspects of dementia, and other current conditions, have been attracting quite a lot of research interest recently [1–11]. In general, utilizing traditional statistical theoretical approaches to determine epidemic/outbreak probability/risk and realistic biological system reliability factors under real-world dementia settings being rather difficult [12–19]. The latter being often brought on by a variety of bio-system degrees of freedom (components), and numerous random variables that affect bio-system’s dynamics. In theory, a complex biological or public health system's reliability may be directly assessed by performing direct MC (Monte Carlo) simulations, or having enough clinical observations. For dementia, however, some of the available observed patient global numbers are limited by the beginning of the year 1990, (https://ourworldindata.org/causes-of-death.). To contribute further dementia prognostic research, authors of this study have developed novel bio-reliability technique, suitable for bio and health systems, enabling accurate future risks forecast. Naturally, the entire world’ s countries were selected, due to availability of online health observations and associated research.

EVT (Extreme value theory) or and statistical lifespan data modeling being widely used in bio-engineering, public health and medicine. There have been numerous recent publications supporting and criticizing upper boundaries distribution of life expectancy [20, 21]. For statistically significant data, studies in these disciplines frequently use a parametric bivariate lifespan distribution derived from the exponential distribution [22]. In [23, 24], authors proposed new approach, that uses Power Variance Function copulas (for example Clayton, Gumbel, inverse Gaussian copulas), conditional sampling, and numerical approximation used in survival analysis.

For general reliability studies, not necessarily limited to bio and public health systems [25–29]. In [30–32] authors used EVT to predict mutation in evolutionary genetics risks, and further develop a likelihood framework from EVT, that was used to determine mutation fitness effects. Similarly, in [33], authors used Beta-Burr distribution, expanding EVT theory to calculate mutation fitness effects. In [22], authors discussed bivariate logistic regression models, which were employed in a cognitive experiment for visual recognition and to access multiple sclerosis fatalities/hazards with walking impairments.

Clinical incidence data for dementia in 195 global nations between 1990 and 2019 has been obtained from a public source, (https://ourworldindata.org/causes-of-death). The bio-system under examination here can be thought of as a multi-degree of freedom (MDOF) dynamic bio-system, with highly inter-correlated regional components/dimensions.

Although this study aimed at forecasting future dementia outbreaks, and not on disease symptoms themselves. Figure 1 presents global world countries map with related dementia cases.

**Fig. 2:**
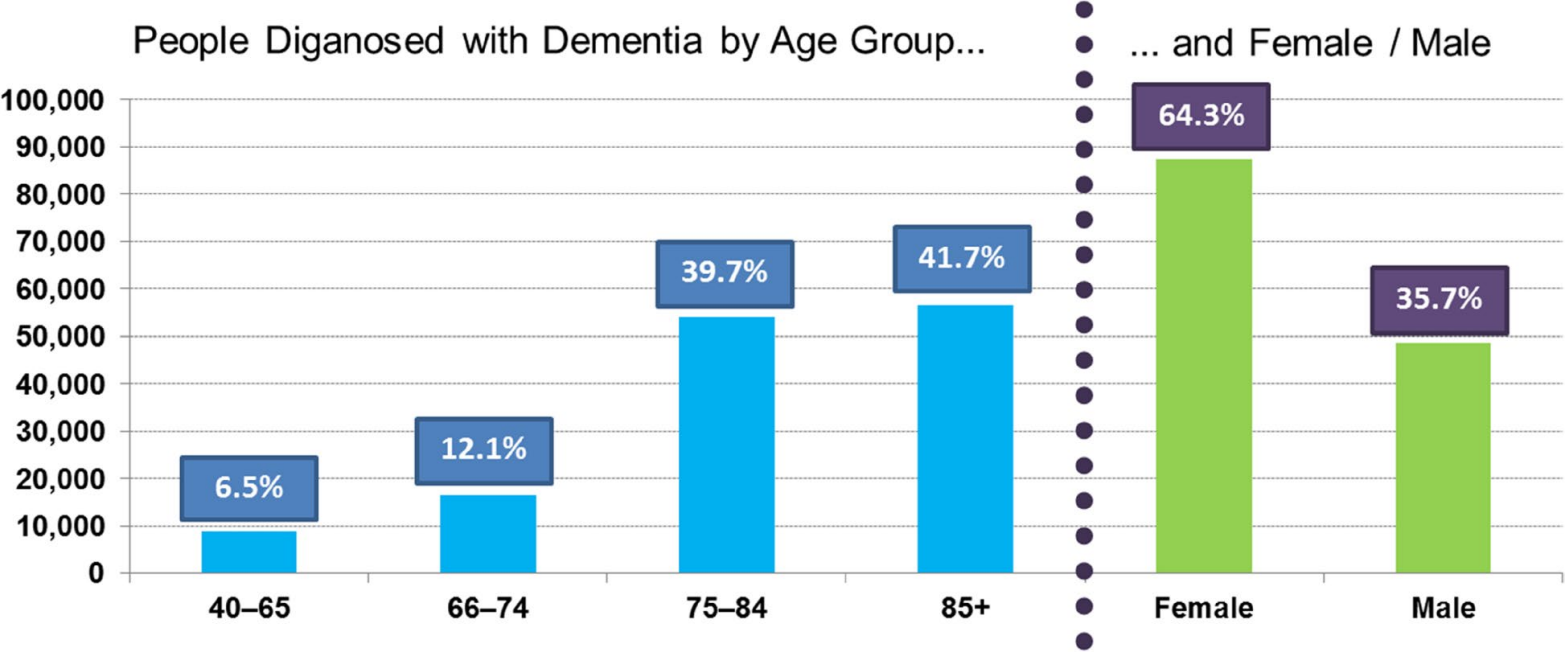
Dementia percentage in Ontario, Canada, by age groups and gender [34]

While dementia is most common among people over 65 years old, almost 7% of dementia diagnoses in Ontario, Canada being made in people aged 40 to 65 years. Additionally, about 64% of Ontario, Canada population that have been diagnosed with dementia, being women, Fig. 2.

## Method

**Fig. 3:**
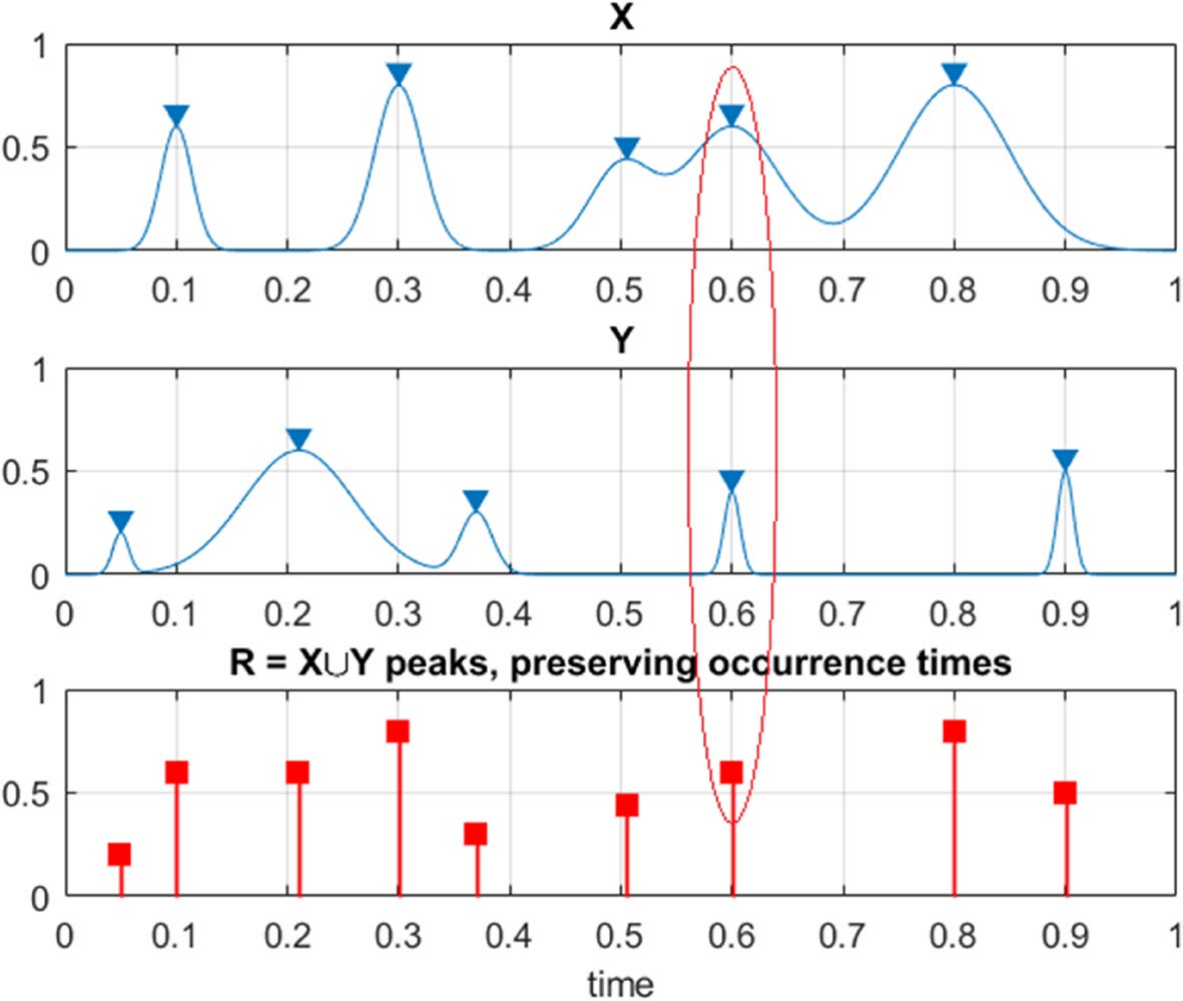
Example of how 2 components, X and Y, being merged to create 1 new synthetic vector $\overrightarrow{R}$. Red ellipse highlights case of simultaneous maxima for 2 different biosystem components

Let one consider multi degree of freedom (MDOF) dynamic bio-system, consisting of its critical system components $X\left(t\right), Y\left(t\right), Z\left(t\right), \dots$, being represented by response/load assembled vector $\left(X\left(t\right), Y\left(t\right), Z\left(t\right), \dots \right)$, being either measured/simulated/observed or simulated over a sufficiently long (representative) time lapse $(0,T)$. Unidimensional biosystem component global maxima being denoted as $X_{T}^{\max}=\max_{0\leq t\leq T}X\left(t\right)$, ${Y}_{T}^{\mathrm{max}}={\mathrm{max}}_{0\le t\le T}Y\left(t\right)$, ${Z}_{T}^{\mathrm{max}}={\mathrm{max}}_{0\le t\le T}Z\left(t\right), \dots$. By sufficiently long (representative) time lapse $T$ authors mean large enough (representative) value of $T$ with respect to the dynamic system auto-correlation and relaxation times. Let ${X}_{1},\dots ,{X}_{{N}_{X}}$ be temporally consequent local maxima of the biosystem component process $X=X(t)$ at discrete temporally increasing times ${t}_{1}^{X}<\dots <{t}_{{N}_{X}}^{X}$ within $(0,T)$. Identical definitions follow for other MDOF components $Y\left(t\right), Z\left(t\right), \dots$ namely ${Y}_{1},\dots ,{Y}_{{N}_{Y}};$ ${Z}_{1},\dots ,{Z}_{{N}_{Z}}$ and so on. For simplicity, all biosystem components, and hence their maxima have been assumed to be non-negative. Then

1 $$P=\iiint\nolimits_{\left(0, 0, 0, , \dots \right)}^{\left({\eta }_{X}, {\eta }_{Y}, {\eta }_{Z }, \dots \right)}{p}_{{X}_{T}^{\mathrm{max}}, { Y}_{T}^{\mathrm{max}}, { Z}_{T}^{\mathrm{max}} , \dots }\left({x}_{T}^{\mathrm{max}}, {y}_{T}^{\mathrm{max}},{ z}_{T}^{\mathrm{max}}, \dots \right)d{x}_{T}^{\mathrm{max}}d{y}_{T}^{\mathrm{max}}d{z}_{T}^{\mathrm{max}}\dots$$

being the probability of dynamic biosystem survival with critical values of system components being denoted as${\eta }_{X}$,${\eta }_{Y}$,${\eta }_{Z}$,…; $\cup$ beings logical unity operator «or»; ${p}_{{X}_{T}^{\mathrm{max}}, { Y}_{T}^{\mathrm{max}}, { Z}_{T}^{\mathrm{max}} , \dots }$ being joint probability density function (PDF) of the individual component maxima. If biosystem number of degrees of freedom (NDOF) being large, it being not practically feasible to estimate directly biosystem’s joint PDF ${p}_{{X}_{T}^{\mathrm{max}}, { Y}_{T}^{\mathrm{max}}, { Z}_{T}^{\mathrm{max}} , \dots }$ and therefore target survival biosystem probability$P$. The latter probability $P$ however, needs to be estimated, as system expected lifetime, according to Eq. (1). Bio-system unidimensional components $X, Y, Z, \dots$ being now re-scaled and non-dimensionalized as follows

2 $$X\to \frac{X}{{\eta }_{X}}, Y\to \frac{Y}{{\eta }_{Y}}, Z\to \frac{X}{{\eta }_{X}}, \dots$$

making all two responses non-dimensional and having the same failure/hazard limit equal to 1. Next, unidimensional system components local maxima being merged into one temporally non-decreasing synthetic vector ${\varvec{R}}\left(t\right)\equiv \overrightarrow{R}=\left({R}_{1}, {R}_{2}, \dots ,{R}_{N}\right)$ in accordance with corresponding merged time vector ${t}_{1}\le \dots \le {t}_{N}$, $N\le {N}_{X}+{N}_{Y}+{ N}_{Z}+\dots$. Each local maxima ${R}_{j}$ being an actual encountered bio-system component local maxima, corresponding to either $X\left(t\right)$ or $Y\left(t\right)$, or $Z\left(t\right)$ or other system components. Constructed synthetic $\overrightarrow{R}$-vector has zero net data-loss, see Fig. 3.

Now the non-decreasing synthetic vector $\overrightarrow{R}$, and it’s corresponding temporally non-decreasing occurrence times ${t}_{1}\le \dots \le {t}_{N}$, have been now fully introduced [35–45].

## Results

In both neurology and mathematical biology, the focus has been on the prediction of dementia-like conditions. Public health dynamics being well-known for its highly non-linear, multidimensional, spatially cross-correlated dynamic bio-system properties, that being challenging to analyse. Different methods have been used in earlier research to mimic dementia death rates. This section uses a novel approach to raw clinical dementia datasets, given as annually recorded time-series for all nations in the world to demonstrate the effectiveness of the aforementioned methodology [16].

**Fig. 4:**
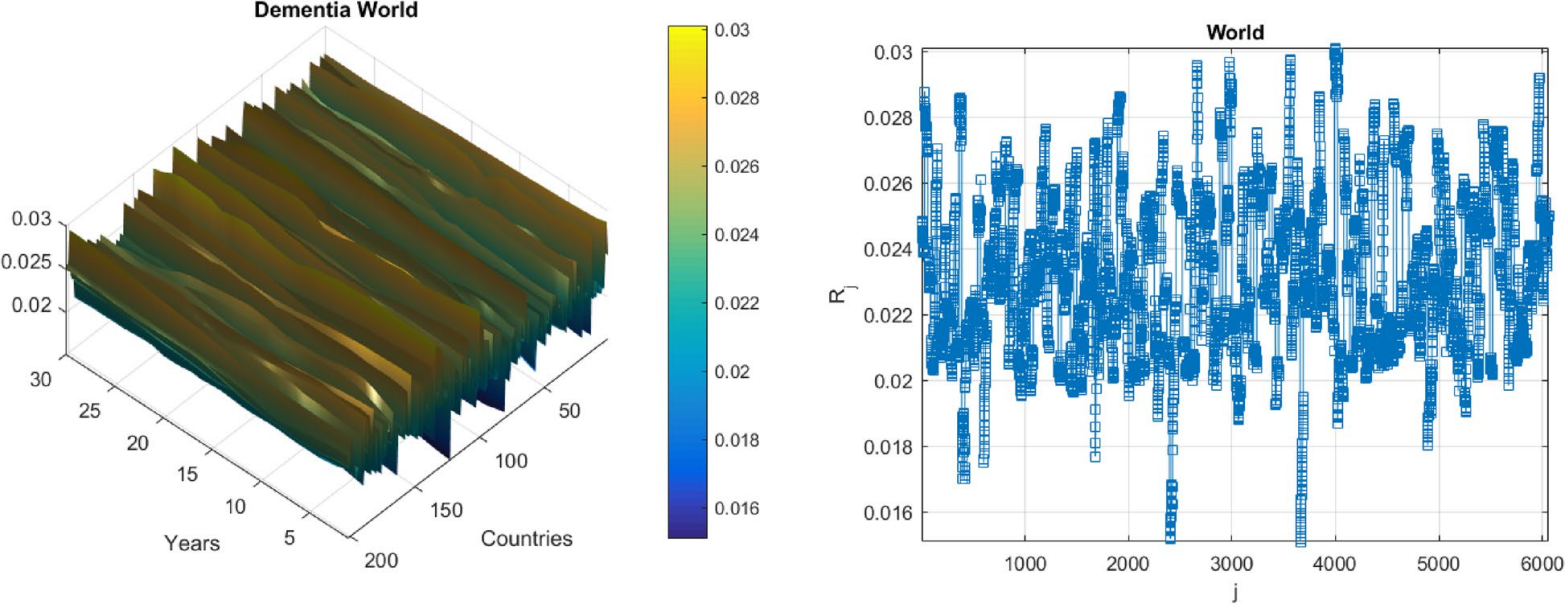
Dementia, Left: 2D surface of annual death cases as % of local population, per country, and per year. Right: Annual recorded death rate in percent as 195D vector $\overrightarrow{R}$. scaled by in percent of corresponding country’s population

The strategy mentioned above being illustrated in practice in this section. The present section's statistics information was obtained from the public website, (https://ourworldindata.org/causes-of-death). This website contains data on dementia death rates worldwide from 1990 to 2020. The component was the number of patients who died in 195 different nations throughout the world, with $X, Y, Z, ...$ constituting one hundred ninety-five dimensional (195D) dynamic bio-system. In order to unify all 195 measured/recorded time series $X, Y, Z,\dots$ the following scaling procedure has been performed, according to Eq. (2), making all 195 responses non-dimensional and having the same failure/hazard limits equal to 1. Failure/hazard limits${\eta }_{X}, {\eta }_{Y}, {\eta }_{Z}, \dots$, (dementia thresholds) being not an obvious choice; most straightforward option would be for various nations to establish failure limitations equal to the relevant nation's population in percent to the local population, thereby making $X, Y, Z, \dots$ equal to annual death rate per country. All local biosystem component’s maxima from 195 measured time series have been merged into one single synthetic time series, by keeping them in time non-decreasing order: $\overrightarrow{R}=\left(\mathrm{max}\left\{{X}_{1},{Y}_{1},{Z}_{1},\dots \right\},\dots ,\mathrm{max}\left\{{X}_{N},{Y}_{N},{Z}_{N},\dots \right\}\right)$ with whole vector $\overrightarrow{R}$ being sorted, according to non-decreasing times of occurrence of these local maxima. Figure 4, left represents dementia annually recorded death cases per each country, and per year.

Figure 4, right represents the number of new annually recorded dementia deaths as a 195D vector $\overrightarrow{R}$, consisting of assembled regional/national new annually recorded death rates, for each corresponding country. Afghanistan and Kiribati data have been excluded from analysis, since they both have been regarded as statistical outliers. Vector $\overrightarrow{R}$ has been assembled of different regional/national components, having different dementia backgrounds. Index $j$ being just a running index of biosystem components local maxima encountered in a non-decreasing temporal sequence [46–48].

**Fig. 5:**
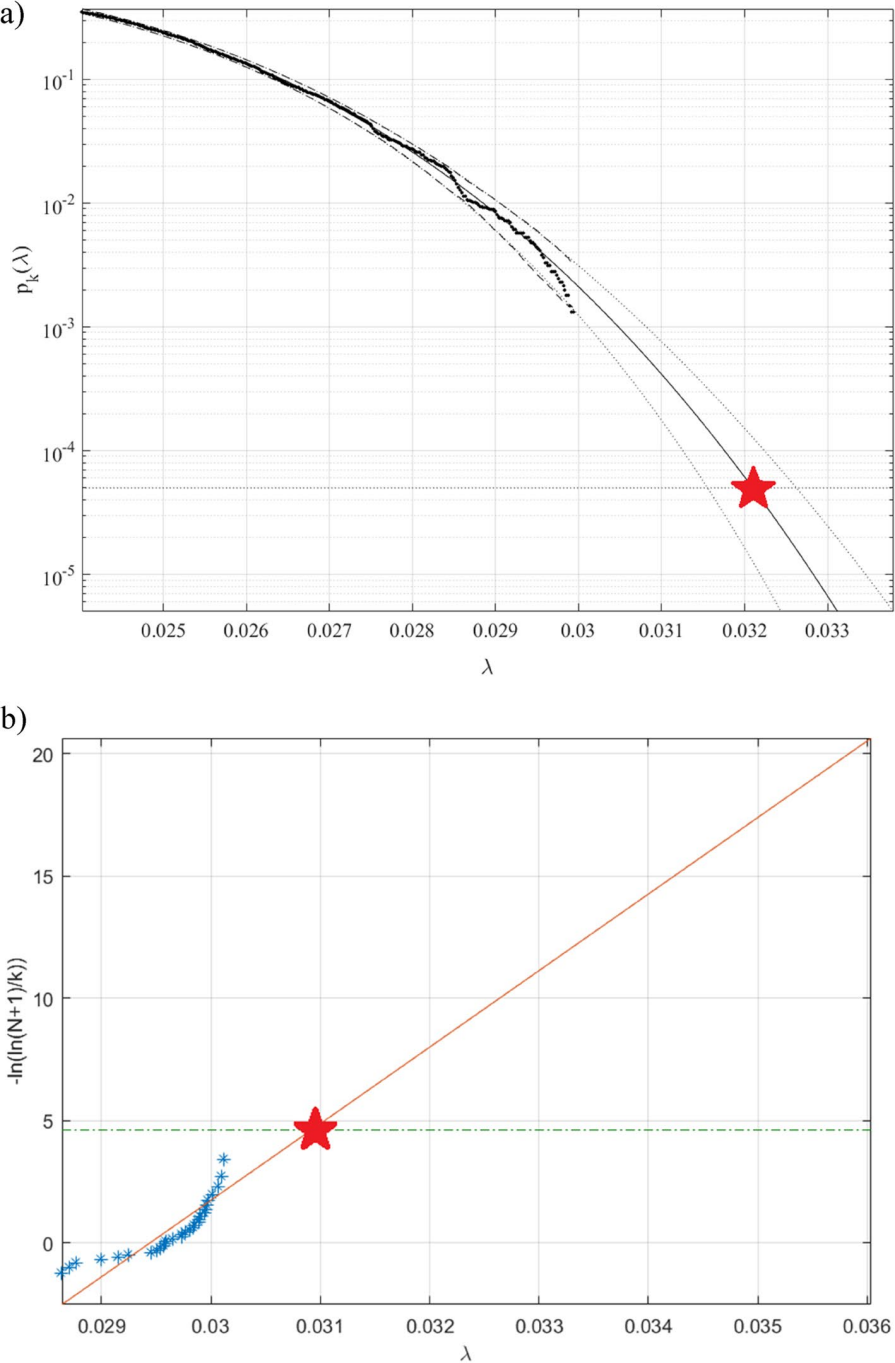
Death rate prediction. **a** 100-years return level extrapolation of ${p}_{k}\left(\lambda \right)$ towards critical level (marked by star), in percent (%) to local population. Extrapolated 95% CI (Confidence Interval) marked by dotted lines. **b** prediction by Gumbel method. Percentage of local population being on horizontal axis

Figure 5 presenting annual death rates (as percentage of dead from dementia to the local/national population per given country) prediction, 100-year return level extrapolation towards dementia outbreak, having 100-year return period, indicated by horizontal dotted line, and somewhat beyond, $\lambda =0.024$% cut-on value has been used, percentage of local population on horizontal axis. Dotted lines indicate extrapolated 95% confidence interval. Biosystem probability $P\left(\lambda \right)$ being directly related to the target biosystem failure/hazard risk $1-P$ from Eq. (1). Thus, bio-system failure/hazard probability $1-P\approx {1-p}_{k}\left(1\right)$ may be now estimated. Conditioning number $k=3$ has been found here to be sufficient, as convergence occurred with respect to $k$, for proofs see [46–48]. Figure 5b) presents reasonably narrow 95% CI, the latter being clear advantage of the proposed methodology. Predicted dementia death rates in any world country, for years to come, e.g., for the next 100-years was found about 0.032% [49–51]. Figure 5a) presents prediction by Gumbel method, it is seen that advocated methodology in Fig. 5b) a) performs significantly more accurate than Gumbel extrapolation method, in terms of CI, however predicted risk levels are very close, namely near 0.03% [35, 52–58].

The 2^nd^-order difference plot (SODP), originated from well-known Poincare plot. SODP provides an efficient QC (quality control) tool for the underlying dataset pattern and quality, based on consecutive differences within time series dataset.

**Fig. 6:**
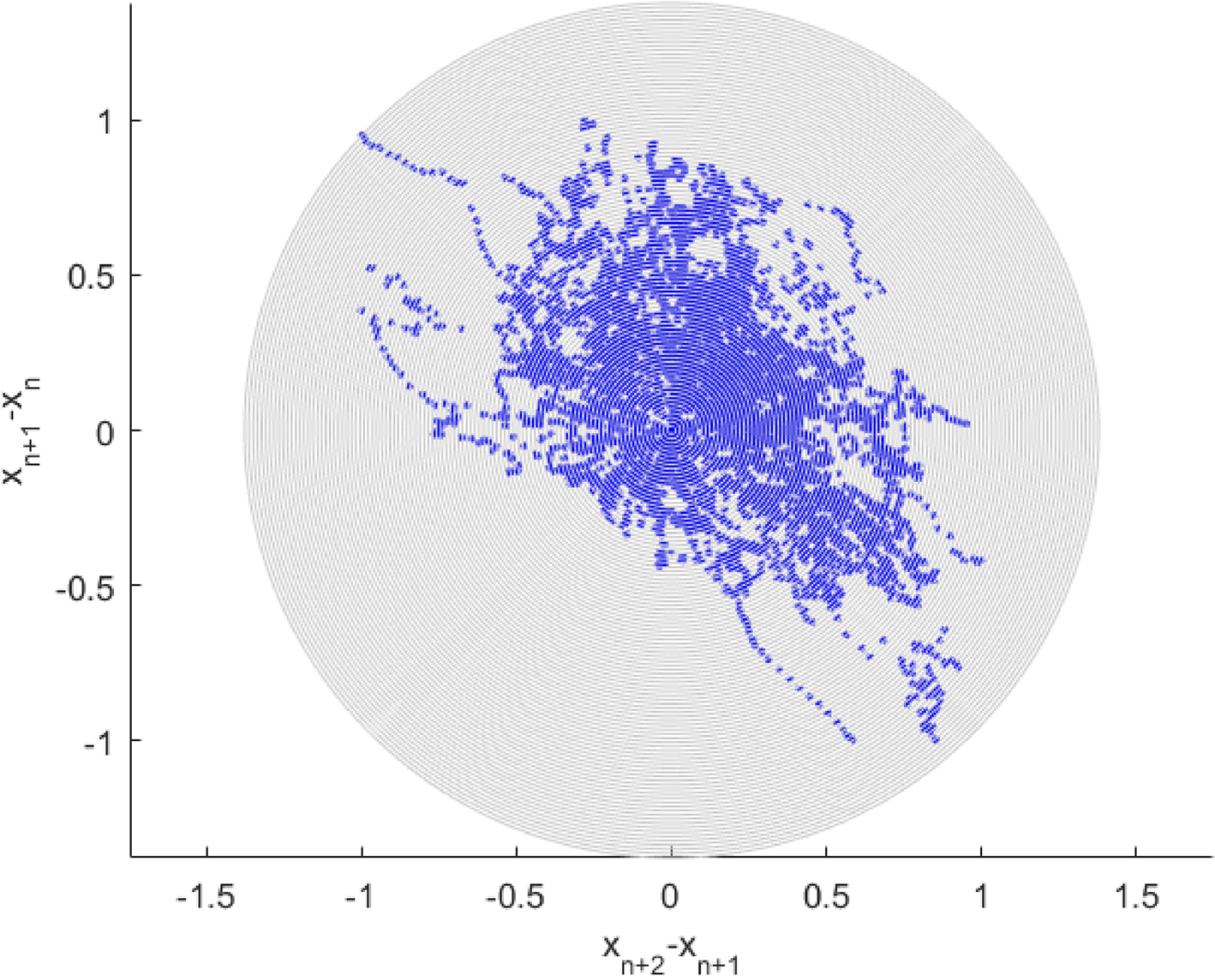
Dementia death global statistics, 2^nd^ order SODP plot

Figure 6 presents 2^nd^-order SODP plot, based on dementia death-rate global statistics, (https://ourworldindata.org/causes-of-death). These plots are often used to identify dataset patterns and compare them with other datasets, especially when using an artificial intelligence (AI) identification method, based on entropy [59]. For review of biomedical-related deep learning, existing concepts, and alternative methods see e.g. [60–63]. Note that while this study introduces MDOF biosystem sub-asymptotic technique, the EVT approach being asymptotic and only 1DOF. The above-described technique, albeit novel, has benefits of effectively utilizing measured raw clinical dataset, yet being able to handle biosystem’s multidimensionality, and conduct accurate extrapolations, based on a relatively small/limited underlying dataset. Note that projected non-dimensional $\lambda$ level, indicated by red star in Fig. 5, represents biosystem probability/risk/hazard of dementia outbreak at any world country, during decades yet to come.

## Discussion

Traditional biological and public health systems reliability techniques, dealing with observed raw clinical time series, do not always cope easily with bio-system’s high-dimensionality, along with nonlinear cross-correlations between different bio-system responses. The key advantage of the presented technique being its ability to analyze bio-reliability of high-dimensional nonlinear dynamic public health systems.

Current study proposes novel multidimensional spatiotemporal modeling technique, and a methodological route to execute forecasting of dementia death rates. It has been explored how to set health system hazard or failure limits appropriately for each nation of interest. This study examined global death rates from dementia, reported across all nations, representing an example of a 195-dimensional (195D) bio-system, observed during three decades 1990—2020. Annual dementia-related death rates constituted multidimensional bio-system’s dynamics in real-time, and has been studied, using the novel reliability approach. The detailed theoretical justification for the suggested strategy has been provided. It should be noted that while use of either direct measurements or Monte Carlo simulations for dynamic biological or public health system’s reliability analysis being appealing, the complexity of bio-system, along with its high dimensionality necessitate development of new, accurate, yet robust techniques, that can handle limited amount of raw clinical data, and make optimal best use of what being already available. The major takeaway being that global public health bio-system being well-managed, generally speaking, if excluding countries like South Korea with alarming dementia growth rates. This study estimated all age categories risk level of 0.032% for the yearly death rate during up to 100-year return period. For ages over 65 years, dementia still poses a potential threat to global health under the current framework for national/regional health management.

A general-purpose, reliable, and simple multidimensional reliability approach was the main goal of this work. The approach described in this study has been previously confirmed by application to a broad variety of simulation models, but only for one-dimensional system responses, and, generally speaking, extremely accurate predictions were achieved. Time series responses can be measured and numerically simulated and analysed. The proposed strategy yielded reasonably narrow confidence intervals, as was demonstrated. As a result, the recommended technique may be useful for a range of reliability investigations on non-linear dynamic biological and public health bio-systems. When addressing relevant age and gender dependencies, prognostics methodology, advocated in this study has wide applicability range, meaning that any specific age ang gender group can be well studied, using advocated methodology. As mentioned, the recommended technique has a wide range of potential applications in public health, thus by no means does the provided dementia example restrict the potential applications of new methodology.

## Data Availability

The datasets generated and/or analyzed during the current study are available in the https://ourworldindata.org/causes-of-death.
